# Development of a Floating Dosage Form of Ranitidine Hydrochloride by Statistical Optimization Technique

**DOI:** 10.4103/0975-1483.71619

**Published:** 2010

**Authors:** S Jain, MS Srinath, C Narendra, SN Reddy, A Sindhu

**Affiliations:** *Department of Pharmaceutics, Government College of Pharmacy, No. 2, P. Kalinga Rao Road, Bangalore-560 027, India*; 1 *Department of Pharmaceutics, VV Pura Institute of Pharmaceutical Sciences, Bangalore-560 070, India*; 2 *Department of Pharmaceutics, M.S. Ramaiah College of Pharmacy, MSR Nagar, MSRIT Post, Bangalore-560 054, India*

**Keywords:** Aerosil, floating lag time, hardness, Ranitidine hydrochloride, 3^2^ factorial design

## Abstract

The objective of this study was to evaluate the effect of formulation variables on the release properties, floating lag time, and hardness, when developing floating tablets of Ranitidine hydrochloride, by the statistical optimization technique. The formulations were prepared based on 3^2^ factorial design, with polymer ratio (HPMC 100 KM: Xanthan gum) and the amount of aerosil, as two independent formulation variables. The four dependent (response) variables considered were: percentage of drug release at the first hour, T_50%_ (time taken to release 50% of the drug), floating lag time, and hardness of the tablet. The release profile data was subjected to a curve fitting analysis, to describe the release mechanism of the drug from the floating tablet. An increase in drug release was observed with an increase in the polymer ratio, and as the amount of aerosil increased, the hardness of the tablet also increased, without causing any change in the floating lag time. The desirability function was used to optimize the response variables, each having a different target, and the observed responses were in accordance with the experimental values. The results demonstrate the feasibility of the model in the development of floating tablets containing Ranitidine hydrochloride.

## INTRODUCTION

Tablets are the most widely used dosage forms because of their convenience in terms of self-administration, compactness, and ease of manufacturing. However, oral administration has only a limited use for important drugs from various pharmacological categories that have poor oral bioavailability, due to incomplete absorption or degradation in the gastrointestinal (GI) tract. Some of these drugs are characterized by a narrow absorption window at the upper part of the gastrointestinal tract. Rapid and unpredictable gastrointestinal transit could result in incomplete drug release from the device above the absorption zone, leading to diminished efficacy of the administered dose.[1] To increase the gastric retention time of drugs, gastroretentive dosage forms (GRDF) can be developed.[2] These systems remain in the gastric region for several hours and can therefore significantly prolong the gastric residence time of the drugs. Prolonged gastric retention improves bioavailability, reduces drug waste, and improves solubility of drugs that are less soluble in the high pH environment of the small intestine.[3,4] It is quite difficult to achieve extensive retention of the GRDF, as the natural activity of the stomach is to evacuate its contents into the intestine. The main approaches that have been examined are low density GRDF that remains buoyant above the gastric fluid; high density, which retains the dosage form in the body of the stomach;[5] concomitant administration of drugs or excipients, which slow the motility of the gastrointestinal tract;[6] and bioadhesive or mucoadhesive dosage forms.[7] As most absorption windows are located in the proximal small intestine (duodenum), the most effective strategy to improve drug absorption will be to retain the formulation in the stomach.[8]

Ranitidine hydrochloride is a histamine H_2_-receptor antagonist. It is widely prescribed in active duodenal ulcers, gastric ulcers, Zollinger-Ellison syndrome, gastroesophageal reflux disease, and erosive esophagitis.[9] The effective treatment of erosive esophagitis requires administration of 150 mg of Ranitidine, four times a day. A conventional dose of 150 mg can inhibit gastric acid secretion up to five hours, but not up to 10 hours. An alternative dose of 300 mg leads to plasma fluctuations; thus a sustained release dosage form of Ranitidine hydrochloride is desirable. The short biological half-life of the drug (~2.5 3 hours) also favors development of a sustained release formulation.

Ranitidine is absorbed only in the initial part of the small intestine and has 50% absolute bioavailability. Moreover, colonic metabolism of Ranitidine is partly responsible for the poor bioavailability of ranitidine from the colon.[10] The gastroretentive drug delivery systems can be retained in the stomach and assist in improving the oral sustained delivery of drugs that have an absorption window in a particular region of the gastrointestinal tract. These systems help in continuously releasing the drug before it reaches the absorption window, thus ensuring optimal bioavailability.

The present study was aimed at developing floating tablets of Ranitidine hydrochloride, using the experimental design technique. A 3^2^ full factorial design was used where the independent / formulation variables determined included a different ratio of polymers (HPMC: Xanthan Gum) and amount of aerosil, while the dependent / response variables determined were drug release in the first hour, time required for 50% of drug release,[11] floating lag time, and hardness of the tablet. A quadratic model was used to quantitatively evaluate the main effects and interaction.

## MATERIALS AND METHODS

### Materials

Ranitidine Hydrochloride was received as a gift sample from Himanshu Pharmaceuticals and Embiotic Laboratories, Bangalore. Hydroxy propyl Methyl Cellulose (HPMC), citric acid, polyvinyl pyrrolidone (PVP), sodium bicarbonate, and colloidal silicon dioxide (Aerosil) were obtained as gift samples from Strides Arcolabs and Zydus Recon, Bangalore. Xantan gum and dicalcium phosphate were obtained as gift samples from Jagath Pharma, Bangalore. Magnesium stearate and talc were received as gift samples from Eros Pvt. Ltd., Bangalore.

### Methods

#### Experimental design

In the present study, a 3^2^ full factorial design containing two factors was evaluated at three levels [Table 1], and the experimental trials were performed in all possible combinations with three replicates of the center point.[12]

**Table 1:** Selected factor levels for the experimental design used in the formulation of floating tablets

Model	Actual values			Coded values		
Factor	Low	Mid	High	Low	Mid	High
Factor A = HPMC: Xanthan Gum (X_1_)	0 : 180	90 : 90	180 : 0	- 1	0	+ 1
Factor B = Aerosil (X_2_)	0	1.5	3.0	- 1	0	+ 1

The two independent formulation variables evaluated were:

X_1:_ Different ratios of polymers (HPMC: Xanthan Gum)

X_2:_ Quantity of aerosil

The response variables evaluated were:

Y_1_: Drug release at the first hour

Y_2_: Time required for 50% of drug release (T_50%_)

Y_3_: Floating lag time (in minutes)

Y_4_: Hardness of the tablet (in kg / cm^2^.)

Preparation of Ranitidine hydrochloride tablets (Preliminary trials)

Formulations were prepared according to the 3^2^ factorial design [Table 2]. The ingredients were passed through a 60 mesh sieve. The required quantities of HPMC, PVP, sodium bicarbonate, xantan gum, and dicalcium Phosphate were blended together in a suitable mixer. Ranitidine Hydrochloride was added to the above mixer in geometrical dilution and mixing was continued. Magnesium stearate, talc, and aerosil were finally added and the blend was then compressed into tablets using 12 mm flat-faced punches in a 10 station rotary tablet machine (Rimek RSB-4 Mini press Cadmach, Ahmedabad, India).

**Table 2:** Composition of floating tablets of Ranitidine hydrochloride

Ingredients	D 1	D 2	D 3	D 4	D 5	D 6	D 7	D 8	D 9	D 10	D 11
Ranitidine Hydrochloride	336	336	336	336	336	336	336	336	336	336	336
HPMC K-100 M	0	90	180	0	90	180	0	90	180	90	90
Xanthan gum	180	90	0	180	90	0	180	90	0	90	90
Aerosil	0	0	0	10	10	10	20	20	20	10	10
PVP K-30	60	60	60	60	60	60	60	60	60	60	60
Sodium bicarbonate	50	50	50	50	50	50	50	50	50	50	50
Dicalcium phosphate	30	30	30	30	30	30	30	30	30	30	30
Magnesium stearate	6	6	6	6	6	6	6	6	6	6	6
Talc	12	12	12	12	12	12	12	12	12	12	12
Total weight	674	674	674	684	684	684	694	694	694	684	684

*All the quantities expressed are in terms of milligrams

## EVALUATION OF TABLET PROPERTIES

### Hardness

The crushing strength of the tablets was measured using a Pfizer hardness tester. Three tablets from each formulation batch were tested randomly and the average reading noted. The readings are given in Table 3.

**Table 3:** Post-compression parameters for designed formulations

Parameters	D1	D2	D3	D4	D5	D6	D7	D8	D9	D10	D11
Hardness (kg / cm^2^)	5.2	6.2	7.0	6.2	6.4	8.8	6.2	8.4	12.6	10.8	7.8
Floating Lag time (min)	1.70	4.39	2.58	1.77	0.81	1.08	1.20	1.19	0.42	1.02	0.82

### Friability

The friability of the tablet was determined using Roche Friabilator (Electrolab). Twenty previously weighed tablets were rotated at 25 rpm for four minutes. The weight loss of the tablets before and after measurement[13] was calculated using the following formula:

$$\mathrm{Percentage}\;\mathrm{friability}\;=\;\frac{\mathrm{Initial}\;\mathrm{weight}\;-\;\mathrm{Final}\;\mathrm{weight}}{\mathrm{Initial}\;\mathrm{weight}}\;\times \;100$$

### Weight variation

The test was carried in conformity with the official method described in I.P (1996).[14] Twenty tablets from each batch were selected randomly after compression, weighed individually, and the average weight was determined. None of the tablets deviated from the average weight by more than 5%.

### Floating lag time

A tablet was placed in a dissolution flask with 400 ml of simulated gastric fluid maintained at 37 ± 1°C. Subsequently, the time taken by tablet to move from the bottom to the top of the flask, in minutes, was measured.[15] The readings are given in Table 3

### Duration of buoyancy

Duration of buoyancy was observed simultaneously when the dissolution studies were carried out. The time taken by the tablet to rise to the surface of the dissolution media and time taken for it to sink was noted, the difference of which gives the duration of buoyancy.[16]

### Drug content

Ten tablets were randomly sampled from each formulation batch, finely powdered and individually estimated for the drug content after suitable dilution, using UV-VIS spectrophotometer (UV-1601, Shimadzu) at 313.5 nm.

### *In vitro* drug release studies

*In vitro* drug release studies for all the formulations were carried out using the tablet dissolution test apparatus (USP TDT 06PL, Electrolab, Mumbai). The dissolution medium used was simulated gastric fluid pH 1.2 (without enzymes) maintained at 37° C and the media was rotated at 50 rpm. Aliquots were withdrawn at 1-hour intervals for 12 hours, filtered and analyzed spectrophotometrically at 313.5 nm for cumulative drug release. The dissolution studies were conducted in triplicates and the mean values were plotted against time.

### Data analysis

To analyze the mechanism of drug release and release rate kinetics from the dosage form, the data obtained were fitted into zero order, first order, Higuchi release and Korsmeyer and Peppas release model using Prism and Sigma plot^®^ software.[17]

### Zero–order release kinetics

To study the zero-order release kinetics, the release rate data are fitted to the following equation:

F = Kt

where, ‘F’ is the fraction of drug release, ‘K’ is the release rate constant and ‘t’ is the release time.

### First-order release kinetics

To study the first-order release kinetics the release rate data are fitted to the following equation:

$$F\;=\;100*\left(1-e^{-\mathrm{Kt}}\right)$$

### Higuchi release model

To study the Higuchi release model the release rate data are fitted to the following equation:

$$F\;=\;{\mathrm{Kt}}^{1/2}$$

### Korsmeyer and Peppas release model

To study the Korsmeyer and Peppas release model the release rate data are fitted to the following equation:

$$M_{t}/M_{\infty }\;=\;K^{\mathrm{tn}}$$

Where, M_t_ / M_∞_ is the fraction of drug release, ‘K’ is the release rate constant, ‘t’ is the release time and ‘n’ is the diffusional exponent for the drug release that is dependent on the shape of the matrix dosage form.

### Statistical analysis

The effect of formulation variables on the response variables were statically evaluated by applying one-way ANOVA at 0.05 level using a commercially available software package Design of Experiments^®^ 6.05 (Stat Ease, USA). The design was evaluated by a quadratic model, which bears the form of the equation:

$$Y\;=\;b_{0}\;+\;b_{1}X_{1}\;+\;b_{2}X_{2}\;+\;b_{3}X_{1}X_{2}\;+\;b_{4}{X_{1}}^{2}\;+\;b_{5}{X_{2}}^{2}$$

Where Y is the response variable, b_0_ the constant, and b_1_, b_2_, b_3_…b_5_ is the regression coefficient. X_1_ and X_2_ stand for the main effect; X_1_ X_2_ are the interaction terms, and show how response changes when two factors are simultaneously changed. X_1_^2^, X_2_^2^ are quadratic terms of the independent variables to evaluate the non-linearity.

## RESULTS AND DISCUSSION

The tablets were prepared following 3^2^ full factorial design. For floating drug delivery system, the polymers used must be highly swellable in the shortest time. Hence, HPMC and Xantan gum were chosen as the main swellable polymers. HPMC was included in the formulation with the intention of adhering the dosage form to the inner wall of the stomach and also possibly to control the release of ranitidine from the dosage form. Hence, the effect of presence or absence of HPMC was considered as one of the independent factors.

The rate of swelling of the polymer depends upon the amount of water taken up by the polymer. Hence sodium bicarbonate was added, which upon contact with hydrochloric acid liberated carbon-di-oxide (CO_2_) that escaped from the dosage form by creating pores, through which water could penetrate into the dosage form resulting in an increase in the rate of wetting of the polymer and a decrease in the time required for the same.

The hardness of all the formulations was in the range 3.0 – 4.0 kg/cm^2^. The percentage friability of all the formulations was found to be not more than 0.6%. In all the formulations, the drug content was found to be uniform among the different batches of tablets, and ranged from 98.38 to 102.49% of the theoretical value. The average percentage deviation for 20 tablets from each batch was within the acceptable pharmacopeial limits.

The floating lag time for formulations containing HPMC and Xanthan gum was found to be between 3 and 30 minutes.

### Kinetic mechanism (curve fitting)

Fitting of the release data to the Krosmeyer and Peppas equation it was found that, the drug release rate at the first hour (%) ranged from 13.31 ± 0.5323 to 27.16 ± 1.087, the diffusion coefficient (n) ranged from 0.5167 ± 0.01913 to 0.6752 ± 0.01744, and the T_50%_ ranged from 3.179 to 6.188 hours. These results indicated that the release mechanism was by diffusion and erosion. The diffusion coefficient values indicated that the drug release followed the non-Fickian transport. The results are tabulated in Table 4.

**Table 4:** Curve fitting data of release profile for designed formulations

Formulations	D 1	D 2	D 3	D 4	D 5	D 6	D 7	D 8	D 9	D 10	D 11
Zero-order release kinetics											
K (h^-1^)	7.442	8.707	9.904	6.371	7.592	9.831	7.215	8.173	9.549	8.715	8.477
SEM	0.4136	0.4198	0.5504	0.2459	0.3296	0.5679	0.2659	0.373	0.5753	0.4229	0.3715
R^2^	0.6563	0.7749	0.6926	0.873	0.8301	0.6363	0.8878	0.8043	0.5919	0.7651	0.8218
First order release kinetics											
K (h^-1^)	0.1279	0.1686	0.2273	0.09621	0.1292	0.2251	0.1172	0.1483	0.2145	0.169	0.158
SEM	0.004828	0.005385	0.006834	0.002553	0.0037	0.00883	0.002911	0.004436	0.00482	0.006122	0.005753
R^2^	0.9466	0.9765	0.9857	0.9745	0.9761	0.9728	0.982	0.9766	0.9899	0.9689	0.9685
Krosmeyer and peppas model											
K (h^-n^)	20.28	21.11	25.89	13.31	16.99	26.98	14.61	19.06	27.16	21.44	19.34
n	0.5365	0.5912	0.5563	0.6605	0.6285	0.5334	0.6752	0.6093	0.5167	0.5843	0.6195
SEM (K)	0.533	0.7371	1.476	0.5323	0.8222	1.37	0.5455	0.7258	1.087	0.5927	0.6797
SEM (n)	0.01252	0.0165	0.02707	0.01871	0.02274	0.02421	0.01744	0.01794	0.01913	0.01307	0.01654
T_50%_ (hr)	5.378	4.3	3.264	7.418	5.571	3.179	6.188	4.869	3.258	4.26	4.634
R^2^	0.9964	0.9951	0.9854	0.995	0.9918	0.9867	0.9959	0.9945	0.9912	0.9968	0.9955
Higuchi model											
K (h^-1/2^)	0.1279	0.1686	0.2273	0.09621	0.1292	0.2251	0.1172	0.1483	0.2145	0.169	0.158
SEM	0.004828	0.005385	0.006834	0.002553	0.0037	0.00883	0.002911	0.004436	0.00482	0.006122	0.005753
R^2^	0.9466	0.9765	0.9857	0.9745	0.9761	0.9728	0.982	0.9766	0.9899	0.9689	0.9685

The response dependent variables such as the drug release at the first hour, time required for 50% of drug release, floating lag time, and hardness were considered. These responses were subjected to multiple regression analyses of variance and the following observations were made.

### Effect of formulation variables on release at the first hour

**Figure 1:**
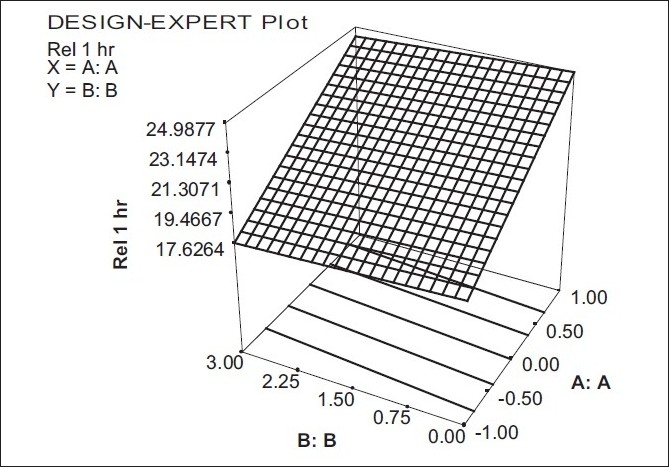
Response surface plot showing the effect of (X_1_) and (X_2_) on the drug release at the first hour (Y_1_)

The model term for Ranitidine hydrochloride release at the first hour was found to be significant with a probability value of 0.0278, indicating an adequate fitting to the surface linear model [Figure 1].

$$Y_{1}\;=\;21.3070\;+\;3.3752\;X_{1}\;-\;0.3054\;X_{2}$$

In this model, factor X_1_, was found to be significant. As the ratio of polymers increased, the amount of drug release at the first hour had increased. Such a behavior of increase in the drug release at the first hour could be attributed to the formation of gel layer with low viscosity of the polymer matrix of HPMC alone, which in turn increased the influx of water into the gel matrix, leading to increased drug diffusion. In this model factor X_2_ was not found to be significant, as the concentration of aerosil did not influence any change in release at the first hour.

### Effect of formulation variables on time required for 50% of drug release

**Figure 2:**
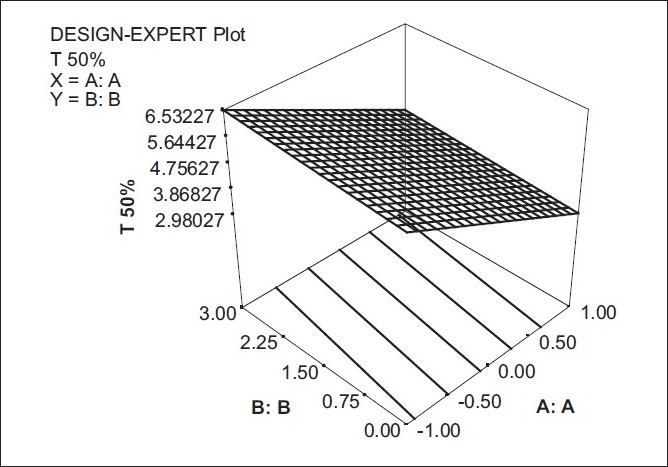
Response surface plot showing the effect of (X_1_) and (X_2_) on time required for 50% of drug release (Y_2_)

The model term for T_50%_ was found to be highly significant with an F value of 0.0008 indicating the adequate fitting of the surface linear model [Figure 2]. As factor A was increased, the ‘T_50%_ ’ values were seen to decrease. However, the amount of aerosil did not show any significant effect on T_50%_.

$$Y_{2}\;=\;4.7562\;-\;1.5471\;X_{1}\;+\;0.2288\;X_{2}$$

### Effect of formulation variables on floating lag time

**Figure 3:**
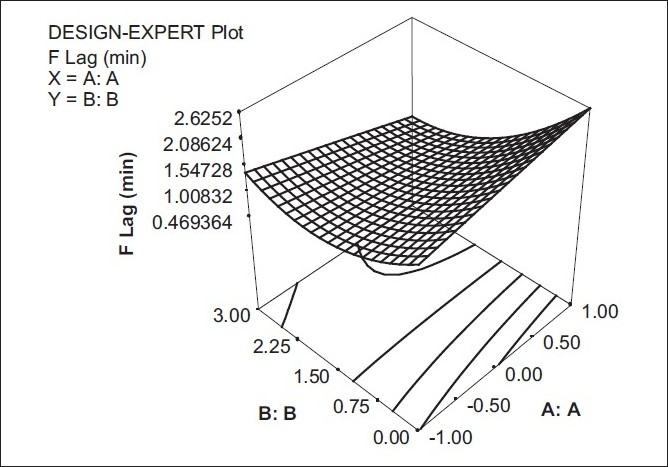
Response surface plot showing the effect of (X_1_) and (X_2_) on Floating Lag Time (Y_3_)

The model term for floating lag time was found to be very significant with the F value of 0.0167, indicating adequate fitting of the quadratic model. As the amount of aerosil in the dosage form increased, the floating lag time decreased, which may be due to the low density of aerosil and also due to the creation of void spaces in the tablet matrix. The factor X_1_ was found to be non-significant on the response floating lag time. The interaction factor X_1_ X_2_ could be studied with the help of response surface plot [Figure 3].

$$Y_{3}\;=\;1.0502\;-\;0.0516\;X_{1}\;-\;0.6616\;X_{2}\;-\;0.0031\;{X_{1}}^{2}\;+\;0.5518\;{X_{2}}^{2}\;-\;0.4162\;X_{1}\;X_{2}$$

### Effect of formulation variables on hardness of the tablet

In this case, the model term for hardness of the tablet was found to be significant, with an F value 0.0135. Both the factors were significantly effective on the hardness of the tablet. As the polymer ratio increased it changed the polymer from Xantan gum to HPMC, and the hardness of a tablet increased. Similarly as the concentration of aerosil increased the hardness of the table also increased.

$$Y_{4}\;=\;7.5636\;+\;1.825\;X_{1}\;+\;1.3666\;X_{2}\;+\;1.1375\;X_{1}\;X_{2}$$

**Figure 4:**
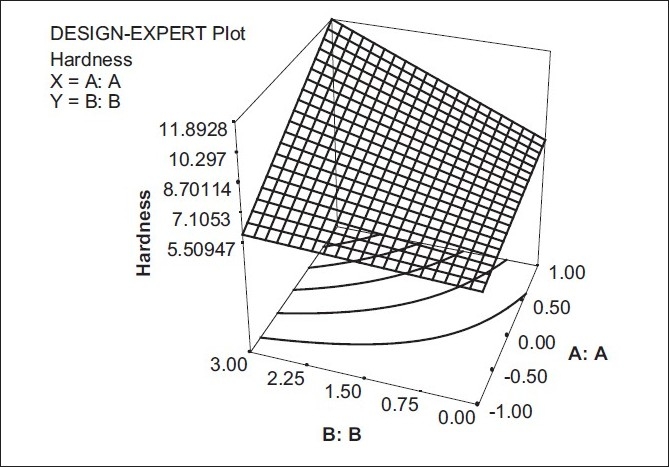
Response surface plot showing the effect of (X_1_) and (X_2_) on the hardness of tablets (Y_4_)

The interaction factor X_1_ X_2_ can be studied with the help of the response surface plot [Figure 4].

The data of pure error and lack of fit are summarized in the ANOVA table [Table 5], which can provide a mean response and an estimate of pure experimental uncertainty. The residual values represent the differences between the observed and predicted values, given that the computed F values are respectively lesser than the critical F value, which denotes non-significance, with regard to lack of fit.

**Table 5:** Summary of ANOVA results in the analysis of lack of fit and pure error

Source	Sum of Squares	DF	Mean Square	F Value	Prob > F
Release at 1^st^ hour					
Model	68.91184	2	34.45592	5.798715	0.0278
X_1_	68.35211	1	68.35211	11.50323	0.0095
X_2_	0.559736	1	0.559736	0.0942	0.7667
Residual	47.53594	8	5.941993	-	-
Lack of Fit	40.86556	6	6.810927	2.04214	0.3647
Pure Error	6.670381	2	3.33519	-	-
Total	116.4478	10	-	-	-
Time required for 50% of drug release					
Model	14.67654	2	7.338268	19.90011	0.0008
X_1_	14.36235	1	14.36235	38.94819	0.0002
X_2_	0.314188	1	0.314188	0.852024	0.3830
Residual	2.950042	8	0.368755	-	-
Lack of Fit	2.037853	6	0.339642	0.744675	0.6704
Pure Error	0.912189	2	0.456094	-	-
Total	17.62658	10	-	-	-
Floating lag time					
Model	4.163918	5	0.832784	8.653103	0.0167
X_1_	0.016017	1	0.016017	0.166422	0.7002
X_2_	2.626817	1	2.626817	27.29415	0.0034
X_1_^2^	2.53E-05	1	2.53E-05	0.000262	0.9877
X_2_^2^	0.771475	1	0.771475	8.016074	0.0366
X_1_ X_2_	0.693056	1	0.693056	7.201255	0.0436
Residual	0.481205	5	0.096241	-	-
Lack of Fit	0.454488	3	0.151496	11.34095	0.0821
Pure Error	0.026717	2	0.013358	-	-
Total	4.645123	10	-	-	-
Hardness					
Model	36.36604	3	12.12201	7.546334	0.0135
X_1_	19.98375	1	19.98375	12.44051	0.0096
X_2_	11.20667	1	11.20667	6.976502	0.0334
X_1_ X_2_	5.175625	1	5.175625	3.221989	0.1157
Residual	11.24441	7	1.606345	-	-
Lack of Fit	3.004413	5	0.600883	0.145845	0.9631
Pure Error	8.24	2	4.12	-	-
Total	47.61045	10	-	-	-

## OPTIMIZATION

**Figure 5:**
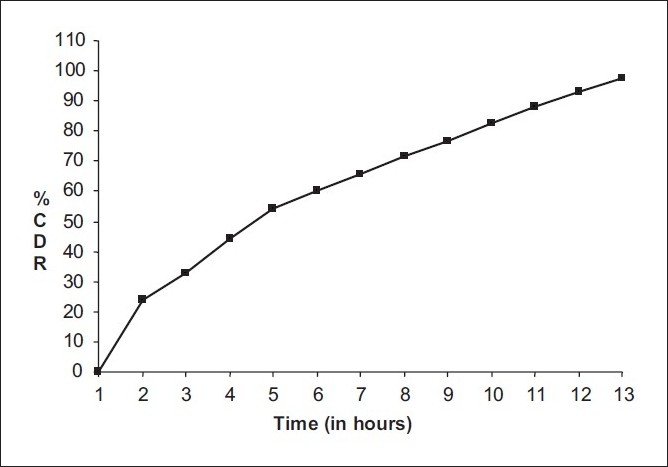
*In vitro* release profile of the optimized formulation

A numerical optimization technique based on the desirability approach was used to generate the optimum settings for the most effective formulation with minimum floating lag time and time required for 50% of drug release. The optimized results obtained are included in Table 6. The results in Table 7 demonstrate a good relationship between the predicted and experimental values, confirming the practicability and validity of the model. The curve fitting data for optimized formulation is presented in Table 8. Furthermore, it is concluded that the mechanism of drug release from the Floating Drug Delivery System follows the non-fickian transport. The *in vitro* release profileof the optimized formulation is shown in Figure 5.

**Table 6:** Composition of the optimized formula

Ingredients	Quantity (mg)
Ranitidine hydrochloride	336
HPMC K-100 M	180
Aerosil	20
PVP K-30	60
Sodium bicarbonate	50
Dicalcium phosphate	30
Magnesium stearate	6
Talc	12

**Table 7:** Comparison chart of the predicted and experimental values for optimized formulation

Dependent variables	Optimized formulation	
	Predicted	Experimental
Release at the first hour (%)	23.73	24.38
T 50% (hr)	3.96	3.43
Floating lag time (min)	0.55	0.47
Hardness (Kg/cm2)	12.2	11.83

**Table 8:** Curve fitting data for optimized formulation

Kinetic models	Optimized formulation
Krosmeyer and peppas model	
K (h^-n^)	23.730
n	0.569
SEM (K)	0.484
SEM (n)	0.010
R^2^	0.998
T _50%_ (hr)	3.963
Higuchi model	
K(h^-1/2^)	27.320
SEM	0.292
R^2^	0.988
Zero order release kinetics	
K(h^-1^)	9.335
SEM	0.478
R^2^	0.734
First order release kinetics	
K(h ^-1^)	0.1967
SEM	0.0067
R^2^	0.9768

## CONCLUSIONS

The present study was an attempt to formulate a gastroretentive floating drug delivery system of Ranitidine Hydrochloride, in order to improve its gastric residence time and bioavailability. A 3^2^ full factorial design was performed to study the effect of formulation variables on drug release at the first hour, time required for 50% of drug release, floating lag time, and hardness of the tablets of Ranitidine Hydrochloride, by applying the optimization technique

The data from the release profile were fitted to various mathematical models, and fitting to the Korsmeyer and Peppas equation revealed that the release mechanism from the dosage form followed the non-fickian transport. Optimization by desirability function was performed to get the optimized formulae and the actual response values were in close agreement with the predicted values, thereby demonstrating the practicability and validity of the model.
